# Cell factories for insulin production

**DOI:** 10.1186/s12934-014-0141-0

**Published:** 2014-10-02

**Authors:** Nabih A Baeshen, Mohammed N Baeshen, Abdullah Sheikh, Roop S Bora, Mohamed Morsi M Ahmed, Hassan A I Ramadan, Kulvinder Singh Saini, Elrashdy M Redwan

**Affiliations:** Department of Biological Sciences, Faculty of Science, King Abdulaziz University, P.O. Box 80203, Jeddah, 21589 Saudi Arabia; Nucleic Acids Research Department, Genetic Engineering and Biotechnology Research Institute (GEBRI), City for Scientific Research and Technology Applications, Alexandria, Egypt; Cell Biology Department, Genetic Engineering and Biotechnology Division, National Research Centre, Tahrir St. Dokki, Cairo, 12311 Egypt; Protein Research Department, Genetic Engineering and Biotechnology Research Institute, City for Scientific Research and Applied Technology, New Borg AL-Arab, Alexandria, Egypt

**Keywords:** Recombinant insulin, *E. coli*, Yeast, Expression system, Posttranslational modifications, Transgenic plants

## Abstract

The rapid increase in the number of diabetic patients globally and exploration of alternate insulin delivery methods such as inhalation or oral route that rely on higher doses, is bound to escalate the demand for recombinant insulin in near future. Current manufacturing technologies would be unable to meet the growing demand of affordable insulin due to limitation in production capacity and high production cost. Manufacturing of therapeutic recombinant proteins require an appropriate host organism with efficient machinery for posttranslational modifications and protein refolding. Recombinant human insulin has been produced predominantly using *E. coli* and *Saccharomyces cerevisiae* for therapeutic use in human. We would focus in this review, on various approaches that can be exploited to increase the production of a biologically active insulin and its analogues in *E. coli* and yeast. Transgenic plants are also very attractive expression system, which can be exploited to produce insulin in large quantities for therapeutic use in human. Plant-based expression system hold tremendous potential for high-capacity production of insulin in very cost-effective manner. Very high level of expression of biologically active proinsulin in seeds or leaves with long-term stability, offers a low-cost technology for both injectable as well as oral delivery of proinsulin.

## Introduction

The pioneering work of Stanley Cohen and Herbert Boyer, who invented the technique of DNA cloning, signaled the birth of genetic engineering, which allowed genes to transfer among different biological species with ease [1]. Their discovery led to the development of several recombinant proteins with therapeutic applications such as insulin and growth hormone. Genes encoding human insulin and growth hormone were cloned and expressed in *E. coli* in 1978 and 1979 respectively. The first licensed drug produced using recombinant DNA technology was human insulin, which was developed by Genentech and licensed as well as marketed by Eli Lilly in 1982.

**Figure 1:**
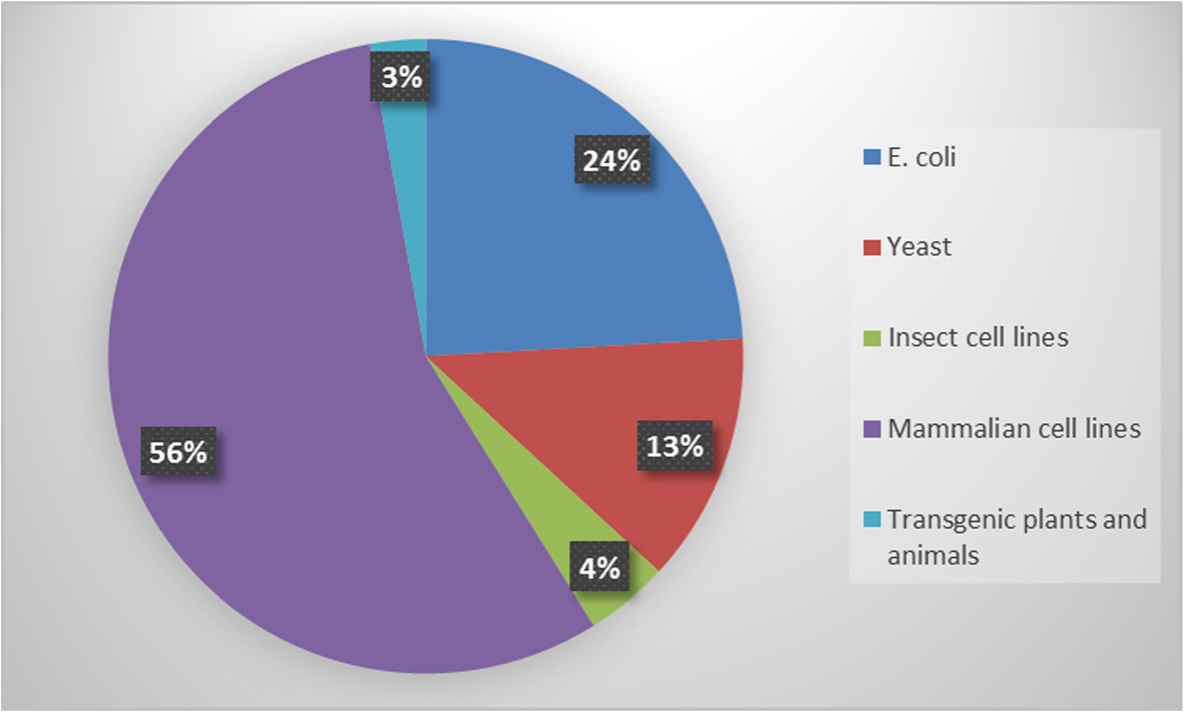
**Percentage of biopharmaceuticals produced in different expression systems** [5-13]**.**

There are more than 300 biopharmaceutical products including therapeutic proteins and antibodies in the market with sales exceeding USD100 billion [2,3]. Therapeutic monoclonal antibodies have captured the major market share (>USD18 billion) followed by the hormones (>USD11 billion) and growth factors (>USD10 billion) [4]. Biopharmaceuticals approved by the US Food and Drug Administration (FDA) and European Medicines Agency (EMA) from 2004 to 2013 are largely derived from mammalian cell (56%); *E. Coli* (24%); *S. Cerevisiae* (13%); Transgenic animals & plants (3%) and insect cells (4%) as shown in Figure 1 [5-13]. At present, insulin is being produced predominantly in *E. coli* and *Saccharomyces cerevisiae* for treatment of diabetic patients.

Since the early 1920s, diabetic patients were treated with insulin, which was purified from bovine or porcine pancreas. The development in the field of genetic engineering allowed the production of insulin in *E. coli* and yeast, which have been approved for therapeutic applications in human by FDA [14,15].

Nowadays, recombinant human insulin is mainly produced either in *E. coli* or *Saccharomyces cerevisiae*. Using *E. coli* expression system, the insulin precursors (IP) are produced as inclusion bodies and fully functional polypeptides are obtained finally by solubilization and refolding procedures [16]. Yeast based expression system yield soluble IP which is secreted into the culture supernatant [17-19]. *Saccharomyces cerevisiae* is the most preferred and predominant yeast for large scale commercial production of insulin, however several other alternate yeast strains have been explored for insulin production [20-24]. Besides, *E.coli* and yeast, mammalian cells, transgenic animals and plant expression systems are also employed as a host for large-scale production of recombinant insulin [25-28].

The incidence of diabetes is increasing at an alarming rate and it has been speculated that the number of diabetic patients worldwide would increase to approximately 300 million by the year 2025 [29]. Consequently, the requirement for insulin will increase manifold (approximately more than 16000 kg/ year) and the productivity of current insulin expression system would not be sufficient to meet the future market demands. Efficient expression systems for insulin production are also needed and novel route for insulin administration such as oral or inhalation are to be developed.

Several recombinant protein based drugs, produced by various expression systems are approved by FDA. Among prokaryotes, *Escherichia coli* has always been preferred for production of recombinant proteins as it offered several advantages including high growth rate, simple media requirement, easy to handle, high yield and very cost effective. However, there are some disadvantages using *E. coli* expression system, such as loss of plasmid and antibiotic property, unsolicited inducers for gene expression, intracellular accumulation of heterologous proteins as inclusion bodies, improper protein refolding, lack of post- translational modifications (including unable to form disulphide bonds), protein-mediated metabolic burden and stress, endotoxin contamination, poor secretion, proteolytic digestion and complexity in downstream process [30-32].

**Figure 2:**
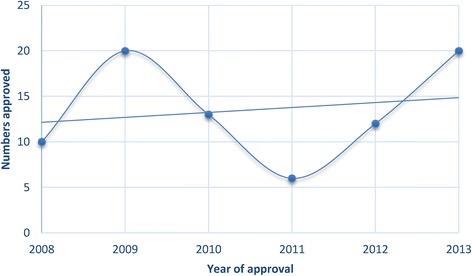
**Approval numbers of biopharmaceuticals in United States and/or European Union over the past six years with a trend line showing the mean approval rate** [5-13]**.**

Among yeast strains, *Saccharomyces cerevisiae, Hansenulla polymorpha and Pichia pastoris* are very commonly used for production of recombinant proteins [21,24,33-35]. Like *E. coli,* they grow rapidly and are very easy to handle and amenable to various genetic manipulations. Recombinant proteins produced in yeast are properly folded and glycosylated to a certain extent similar to the one expressed in mammalian cells. Various human therapeutic proteins, including therapeutic monoclonal antibodies are being produced in mammalian cell lines such as Chinese hamster ovary (CHO) and Baby hamster kidney (BHK) cells. Recombinant proteins expressed in mammalian cells are properly folded, glycosylated and generally yield a functionally active protein [36]. However, the production cost of biopharmaceuticals using mammalian expression system is very high due to expensive culture media. However, when we look at the number of approved biopharmaceuticals by United States and/or the European Union for 2013 recorded above average as compared to past five years. The mean rate of approval is 13 as shown in the Figure 2 [5-13]. Remarkably, the number is same for both the years 2009 and 2013.

### Structure and function of insulin

The human insulin is comprised of 51 amino acids and has a molecular weight of 5808 Da. It is produced by beta cells of the pancreas and plays a key role in regulating carbohydrate and fat metabolism in the body. Insulin is synthesized as a single polypeptide known as preproinsulin in pancreatic beta cells. Preproinsulin harbours a 24-residue signal peptide, which directs the nascent polypeptide to the endoplasmic reticulum. The signal peptide is cleaved as the polypeptide is translocated into the human of the endoplasmic reticulum resulting in the formation of proinsulin. In the Endoplasmic reticulum, the proinsulin is folded in proper confirmation with the formation of 3 disulphide bonds. Folded proinsulin is then transported to the trans-Golgi network, where it is converted into active insulin by cellular endopeptidases called as prohormone convertases (PC1 and PC2) and exoprotease carboxypeptidase E. The endopeptidases cleaves at two positions, resulting in the release of a fragment termed as C-peptide. The mature insulin, thus formed consists of an A-chain with 21 aminoacids and a B-chain containing 30 aminoacids and both polypeptides linked together by two disulphide bonds. Besides, the A-chain has an intrachain disulphide bond [37,38].

### *E. coli* expression system for production of insulin

*E. coli* is a preferred microorganism for large-scale production of recombinant proteins. However, several disadvantages limit its use for production of recombinant biopharmaceuticals. Various post-translational modifications (PTMs) such as glycosylation, phosphorylation, proteolytic processing and formations of disulfide bonds which are very crucial for biological activity, do not occur in *E. coli* [39,40]. N-linked glycosylation is the most common posttranslational modification of proteins in eukaryotes. It has been discovered that the bacterium *Campylobacter jejuni* possess the capability to glycosylate the proteins and it was also shown that a functionally active N-glycosylation pathway could be transferred to *E. coli* [41]. Although the structure of bacterial N-glycan is different from that observed in eukaryotes, engineering of *Campylobacter* N-linked glycosylation pathway into *E. coli,* provides an opportunity to express heterologous proteins in glycosylated form in *E. coli.* Expression of Pglb oligosaccharyltransferase or (OTase) from *C. jejuni* in *E. coli* showed a significant increase in glycopepetide yield [42,43]. Recently efforts has been made to produce glycosylated proteins with substrates other than native and non-native to *E. coli* and *C.jejuni* [44-48].

The codon usage of the heterologous protein also plays a major role in determining the expression level of recombinant protein. If the codon usage of heterologous protein differs significantly from the average codon usage of the *E. coli* host, it could result in very low expression. Usually, the frequency of the codon usage reflects the abundance of their corresponding tRNA. Therefore, significant differences in codon usage could result in premature termination of translation, misincorporation of aminoacids and inhibition of protein synthesis [49]. Expression of heterologous proteins in *E. coli* can be improved by replacing codons that are rarely found in highly expressed *E. coli* genes with more favorable major codons. Similarly, co-expression of the genes encoding for a number of the tRNA for rare codon, may enhance the expression of heterologous proteins in *E. coli.* There are some commercial *E. coli* strains available that encodes for tRNA for rare codons such as BL21 (DE3) CodonPlus-RIL, BL21 (DE3) CodonPlus-RP (Stratagene, USA) and Rosetta (DE3). BL21 (DE3) CodonPlus-RIL harbors tRNA genes for rare codons like AGG, AGA (arginine), AUA (isoleucine) and CUA (leucine). Similarly, Rosetta (DE3) strain harbors tRNA genes for rare codons like AGG, AGA (arginine), CGG (arginine), AUA (isoleucine), CUA (leucine), CCC (proline) and GGA (glycine). These rare codons have been associated with low expression of proteins in *E. coli,* hence application of these genetically engineered *E. coli* host strains may improve the expression level of heterologous proteins and thus might result in higher yield of desired protein [50-52]. The use of protease-deficient *E. coli* strains, which carry mutations that eliminate the production of proteases may also improve the yield of recombinant protein by reducing proteolytic degradation. *E. coli* strain BL-21, is deficient in two proteases encoded by the *lon* (cytoplasmic) and *ompT* (periplasmic) genes. Rather than the external parameters, targeted methods such as modifications in protease or secretion pathways can provide the insight into biology of recombinant proteins [53]. In *E. coli,* complex and large therapeutic proteins can be secreted in periplasm as it provides an oxidizing environment and help in forming disulphide bonds, which facilitate the proper folding of recombinant proteins and likely to yield reliable N- terminus of expressed protein [54]. Periplasm has advantages over cytoplasm in less protein concentration and proteolytic activity, improve the production titer [55], and enhance the solubility of recombinant protein. Altogether, with these advanced modifications and developments ease the process of target protein production thus accelerating the drug development [56].

Heterologous proteins generally accumulate in *E. coli* as inclusion bodies, which comprise of insoluble misfolded aggregates of proteins. Use of molecular chaperones may increase the protein solubility and assist in proper folding of recombinant protein. Some of the chaperones prevent aggregation of protein and some assist in refolding and solubilization of misfolded proteins. The most important chaperones in E. coli are GroEL, GroES, DnaK, DnaJ, GrpE and Trigger factor. These chaperones may be used singly, or in combination to enhance the protein solubility in *E. coli* [57,58].

Recombinant human insulin was first produced in *E. coli* by Genentech in 1978, using a approach that required the expression of chemically synthesized cDNA encoding for the insulin A and B chains separately in *E. coli* [59]. After expressing independently, the two chains are purified and co-incubated under optimum reaction conditions that promoted the generation of intact and bioactive insulin by disulphide bond formation. The first commercial recombinant insulin was developed for therapeutic use in human by this two-chain combination procedure [60]. Another approach involves the expression of a single chemically synthesized cDNA encoding for human proinsulin in *E. coli* followed by purification and subsequent excision of C-peptide by proteolytic digestion. This approach was more efficient and convenient for large scale production of therapeutic insulin as compared to the two chain combination approach and has been used commercially since 1986 [60]. Eli Lilly followed this technology to produce Humulin, the first recombinant insulin approved in 1982, for the treatment of diabetic patients. These first generation recombinant insulins have an amino acid sequence identical to native human insulin and are preferred over animal derived insulin products [14]. However, advancement in the field of genetic engineering and development of technology to chemically synthesize genes with altered nucleotide sequence, facilitated the development of insulin analogues with altered amino acid sequence. It had been observed that native insulin in commercial preparations usually exist in oligomeric form, as zinc-containing hexamer due to very high concentration, but in blood, biologically active insulin is in monomeric form [61]. Hence, this oligomeric complex should dissociate so that insulin can be absorbed from the site of injection into the blood. Due to this, subcutaneously injected recombinant insulin usually have a slow onset with peak plasma concentration after 2 hours of injection and longer duration of action that last for 6–8 hours [62]. Hence, in order to develop a fast- acting insulin analogue, it was required to modify the amino acids residues whose side chains are involved in dimer or oligomer formation. It has been shown that amino acids residues in insulin B-chain particularly B8, 9,12, 13, 16 and 23-28 play critical role in oligomerization [63,64]. Lispro, developed by Eli Lilly, was the first fast acting insulin analogue to obtain regulatory approval in 1996, for therapeutic use [60]. Insulin Lispro is engineered in such a way that it has similar amino acid sequence as the native insulin but has an inversion of proline-lysine sequence at position 28 and 29 of the B-chain, which resulted in reduced hydrophobic interactions and thus prevented dimer formations. For commercial production of insulin Lispro, a synthetic cDNA encoding for Lys ^B28^- Pro ^B29^ human proinsulin was expressed in *E. coli* and insulin Lispro was excised proteolytically from the proinsulin by treating with trypsin and carboxypeptidase. Another rapid-acting insulin analogue, produced in *E. coli* is Glulisine (Apidra) which was developed by Aventis Pharmaceuticals and approved by US regulatory authorities in 2004. Insulin Glulisine have been generated by replacing B3 asparagine by a lysine and B29 lysine replaced by glutamic acid [14].

To avoid multiple injection, long-acting insulin analogues with prolonged duration of actions have also generated. Insulin Glargine is one of such long-acting insulin analogues, which was developed by Aventis Pharmaceuticals and approved by regulatory authorities of USA and EU in 2000. Insulin Glargine was generated by replacing the C-terminal asparagine of the A-chain with a glycine residue and the C-terminal of the B- chain was modified by adding two arginine residues. These modifications resulted in increase of the isoelectric point (pI) from 5.4 to neutral values. Glargine was produced as proinsulin and expressed in *E. coli* and was finally formulated at pH 4 in soluble form. However, after subcutaneous administration, it precipitated due to neutral pH in the subcutaneous tissue. Resolubilization of insulin occur slowly, resulting in longer duration for its release in the blood [14].

### Yeast expression system for the production of insulin

Yeast is a preferred host for expression of various heterologous proteins that require post-translational modifications for its biological activity. Yeast cell has the ability to carry out numerous post-translational modifications such as phosphorylation, O-linked glycosylation, N-linked glycosylation, acetylation and acylation. Recombinant proteins are expressed in soluble form in yeast and properly folded in functionally active form. Production of biopharmaceuticals using yeast expression system is also very cost effective and is amenable to scale up using large bioreactors. However, one major concern for producing therapeutic glycoprotein for human application is that yeast N-glycosylation is of the high-mannose type, which confers a short half-life in vivo and hyper–immunogenicity and thus render the therapeutic glycoprotein less effective. Various attempts have been made to humanize yeast N-glycosylation pathways in order to produce therapeutic glycoproteins with humanized N-glycosylation structure [65].

The therapeutic proteins produced in yeast are specifically from *Saccharomyces cerevisiae* and include hormones (insulin, insulin analogues, non-glycosylated human growth hormone somatotropin, glucagon), vaccines (hepatitis B virus surface antigen), uprate oxidase from *Aspergillus flavus*, granulocyte-macrophage colony stimulating factor, albumin, hirudin of *Hirudo medicinalis* and human platelets derived growth factor [34]. Like *E. coli*, yeast derived recombinant biopharmaceuticals majorly intended as therapeutics for infectious diseases or endocrine, metabolic disorders. Alternate yeast strains, besides *S. cerevisiae,* are being explored for large-scale production of biopharmaceuticals. Specifically, *Pichia pastoris* has the ability to attain high cell densities by its robust methanol-inducible alcohol oxidase 1 (AOX1) promoter and simple developmental approaches contribute to high quality and quantity of recombinant proteins production. In comparison to *Saccharomyces cerevisiae*, *Pichia pastoris* provides a major advantage in the glycosylation of secreted proteins because it does not hyperglycosylate the heterologous proteins. Both yeast strains have a majority of N-linked glycosylation of the high-mannose type, but the length of the oligosaccharides chain added to proteins in *Pichia* (around 8–14 mannose residues per side chain) is much shorter than those expressed in *Saccharomyces cerevisiae* (approximately 50–150 mannose residues per side chain), suggesting that glycoproteins produced in *Pichia pastoris* may be more suitable for therapeutic use in humans [66,67]. Moreover, very high level of expression of heterologous proteins can be attained in *Pichia pastoris*, that might constitute about 30% of total cellular protein which is very high as compared to *S. cerevisiae* [68,69]. Therefore, *Pichia pastoris* can be an attractive alternate for large-scale production of recombinant insulin and insulin analogues. Comparing the different insulin production systems where the bacterial expression systems show higher average specific productivity and maximum biomass concentrations are higher in yeast, the overall production space-time yield remains similar as shown in Table 1 [70].

**Table 1:** **Comparison of human insulin production systems** [70]

**Source**	***E. coli***	***E. coli***	***S. cerevisiae***	***P. pastoris***
Destination of product	Cytoplasm	Secreted	Secreted	Secreted
Biomass cell dry weight (g/l)	80, in bioreactor with fed-batch culture	1.2, in shake flask with batch culture	5, in shake flask with batch culture	59, in bioreactor with fed-batch culture
Typical spec. growth rate (1/h)	0.08– 0.12	not specified	< 0.33	<0.03
Typical spec. production rate (mg/gh)	14.2	3.4	0.21	0.375
Product concentration (g/L)	4.34	0.009	0.075	3.075
Productivity (mg/l h)	1,085	4.01	1.04	17
Reference	[71]	[72]	[19]	[73]

*Saccharomyces cerevisiae* has been extensively used to produce recombinant human insulin since early 1980s [17,18] and a large proportion of recombinant commercial insulins are produced by this yeast expression system [19,74]. For efficient expression and secretion of recombinant proinsulin in yeast, insulin construct was engineered to contain the native A-chain and a B-chain lacking the C-terminal B30 threonine, either directly fused or linked via a short synthetic C peptide (like AAK). The cDNA sequence encoding for this construct was fused with α-factor signal sequence of *Saccharomyces cerevisiae* for secreted expression of proinsulin which gave yield upto 80 mg/ml of insulin. The single chain proinsulin was purified and converted to active insulin by a trypsin-mediated transpeptidation reaction in presence of threonine ester [19]. Besides native recombinant insulin, various insulin analogues are also being produced in *S. cerevisiae.* Insulin Aspart is another fast-acting insulin analogue, which was produced in *S. cerevisiae*, developed by Novo Nordisk and approved by US FDA in 2001 for therapeutic use in human. Insulin Aspart was generated by replacing proline residue at position 28 with aspartic acid in the B-chain. This genetic modification resulted in an increase in inter-chain charge repulsion, decrease in self-association and thus causing rapid entry into the blood from the site of subcutaneous injection [63,75].

Insulin Detemir is another recombinant long-acting insulin analogue that was commercially produced in *S. cerevisiae,* developed by Novo Nordisk and approved for therapeutic use in human in 2004 by European regulatory authorities. Recombinant Detemir have been generated by removing the threonine residue at the 30 position of the B-chain, and a C14 fatty acid chain covalently attached to the lysine residue at the 29 position of the B-chain. These genetic alterations resulted in the binding of insulin to albumin in plasma, which ensured the slow and constant release of insulin and thus prolonging its duration of action up to 24 hours [76-78].

*Sacharomyces cerevisiae* has been reported for the production of more than 40 different recombinant proteins [79]. A few of which related to diabetes are illustrated in Table 2, along with different characteristics. Few proteins secreted extracellularly by *Sacharomyces cerevisiae* with α-factor leader sequence being repeatedly used for adequate production of recombinant proteins. Furthermore, a synthetic leader sequence had been developed by Kjeldsen and associates at Novo Nordisk for more efficient protein secretion in yeast [79,80].

**Table 2:** **Some of the biopharmaceuticals produced by** ***S. cerevisiae*** [2]

**Type**	**Protein**	**Therapeutic application**	**Leader sequence**	**Titer**
Hormones	Insulin Precursor	Diabetes	Synthetic	80 mg/L
	Glucagon	Diabetes	α-Factor	17.5 mg/L

### Transgenic plants as host for insulin production

Transgenic plants have been utilized to produce recombinant proteins because of their advantage of cost effectiveness, high quality protein processing, absence of human pathogens, ease of production and presence of eukaryotic machinery for posttranslational modifications. Initially, the human growth hormone was the recombinant protein product extracted from transgenic tobacco plant [81]. After that, numerous different products have developed from plants such as Hepatitis-B-Virus surface antigen, antibodies, industrial proteins and milk proteins.

Recombinant human insulin has been successfully expressed and produced in oilseeds of plant *Arabidopsis thaliana* [27]. This technology involved the targeted expression of insulin in subcellular organelles known as oilbodies that allowed very high level of expression with easy recovery of recombinant insulin. Oilbodies are storage organelles inside the oilseeds, which comprises of hydrophobic triacylglycerol core encapsulated by phospholipid membrane and an outer wall of proteins known as oleosins. Genetically engineered oil seeds have been generated with recombinant protein specifically targeted to oilbodies as oleosin fusion [27,82,83]. Then the oilbodies are easily separated from other seed components by liquid-liquid phase separation, which reduced the number of chromatography steps required to obtain purified insulin. It has been observed that insulin accumulated to high level in transgenic seed (0.13% of total seed protein). Recombinant insulin was cleaved from the oleosin fusion partner and matured with trypsin digestion following oil body purification to yield a biologically active insulin. This study clearly demonstrated that expression of insulin as oleosin fusion protein in plant allow accumulation of large amount of recombinant insulin within the seed and also provide simple downstream purification by centrifugation i.e. oilbody purification. Subsequent maturation to obtain biologically active insulin can be accomplished using standard enzymatic methods currently used for commercial production of insulin from *E. coli* and yeast. Oilseeds also act as a natural cellular warehouse, where recombinant insulin can be stockpiled until required [27].

In another approach, transgenic plants have been generated, in which, tobacco and lettuce chloroplasts were transformed with human proinsulin comprised of A, B and C-chains fused with the cholera toxin B subunit [28]. It has been observed that, old tobacco leaves accumulated proinsulin upto 47% of total leaf protein and similarly, old lettuce leaves amassed proinsulin up to 53% of total leaf protein. Proinsulin stored in leaves of lettuce was found to be very stable as up to 40% of proinsulin was detected even in senescent and dried leaves as shown in Table 3. Proinsulin from tobacco leaves was extracted with 98% purity and cleaved by Furin protease to release insulin peptides. Oral delivery of unprocessed proinsulin encapsulated in plant cell or by injection into mice revealed lowering of blood glucose levels similar to commercially available insulins. Based on the yield (3 mg of proinsulin/gm of leaves), it was estimated that one acre of tobacco plantation could yield upto 20 million daily doses of insulin per year [28]. C-peptide of proinsulin, which is not present in current commercially available insulin and insulin analogues derived from *E. coli* and *S. cereviciae*, would be a great advantage in long-term treatment of diabetic complications such as stimulation of nerve and renal functions. Very high level of expression of biologically active proinsulin in tobacco and lettuce leaves and long-term stability in dried leaves offers a reliable low-cost technology for both injectable as well as oral delivery of proinsulin.

**Table 3:** **CTB cholera toxin B subunit proinsulin expression in tobacco and lettuce chloroplasts** [28]

**Type of transgenic plant**	**Tobacco**	**Lettuce**
Destination of the product	Chloroplast	Chloroplast
Percentage of Total leaf protein	47%	53%
Protein yield per gram of leaf tissue	2.92/gm	3.28/gm
Reference	[28]	

## Conclusion

Over the next 20 years, WHO has estimated that insulin sale would grow from $12 billion to $54 billion globally. Dietary and lifestyle changes are causing dramatic increase in diabetes incidence all over the world. Both Type I and Type II diabetic patients use insulin, however late stage Type II diabetes patients require large doses of insulin as they develop insulin resistance. The dramatic increase in the number of diabetic patients globally and exploration of alternate insulin delivery methods such as inhalation or oral route is bound to escalate the demand for recombinant insulin in near future. Current manufacturing technologies will not be able to meet the growing demand of insulin due to limitation in production capacity and high production cost. Recombinant human insulin is produced predominantly using *E. coli* and *Saccharomyces cerevisiae* for therapeutic use in human. However, there is an upmost need to increase the production by several fold of a biologically active insulin and its analogues from *E. coli* and yeast using latest novel and efficient technologies. Another strategy, using a different expression host other than *E. coli* and *Saccharomyces cerevisiae* could be employed. Plant-based expression system hold tremendous potential for high-capacity production of insulin in very cost-effective manner. Very high level of expression of biologically active proinsulin in seeds or leaves with long-term stability, offers a low-cost technology for both injectable as well as oral delivery of proinsulin. Moreover, transgenic seeds can also act as warehouse where recombinant insulin can be stockpiled until required.
